# Network analysis of meaning in life and depressive symptoms in Chinese adolescents

**DOI:** 10.1097/MD.0000000000044762

**Published:** 2025-09-19

**Authors:** Xiubin Wang, Nengzhi Jiang

**Affiliations:** a School of Psychology, Shandong Second Medical University, Weifang, Shandong Province, China.

**Keywords:** adolescents, depressive symptoms, meaning in life, network analysis

## Abstract

Adolescent depressive symptoms are highly prevalent in China, with prior studies highlighting a significant association between meaning in life and depressive symptoms. However, the roles of the presence of meaning (PML) and the search for meaning (SML) with depressive symptoms remain inconsistently defined. This study employed network analysis to explore the associations between PML, SML, and specific depressive symptoms in a sample of 2253 Chinese adolescents (968 males; mean age = 15.00 years). Participants completed the Chinese Meaning in Life Questionnaire and a short Chinese version of the Center for Epidemiologic Studies Depression Scale. Findings revealed that both PML and SML were negatively associated with depressive symptoms. Notably, symptoms such as lack of positive affect and activity retardation were directly connected to both PML and SML. Furthermore, adolescents with lower PML levels demonstrated a more densely interconnected depressive symptom network. These results elucidate the associations between different dimensions of meaning in life and the network structure of adolescent depressive symptoms, offering insights that may inform future interventions.

## 1. Introduction

Depressive symptoms are important negative indicators of mental health, particularly during adolescence when incidence rates are high.^[1]^ A meta-analysis indicated that the global prevalence of self-reported depression among adolescents is 6.1%, while the rate of depressive symptoms detected in Chinese adolescents stands at 14.8%.^[2,3]^ These symptoms have a profound impact on adolescents’ physical and mental health.^[4]^ Depressive symptoms have been associated with multiple factors, with meaning in life consistently emerging as a significant protective cognitive factor. Rooted in existentialist thought, the experience of meaning provides individuals with a sense of direction and purpose, which can help counteract feelings of despair and hopelessness often associated with depression.^[5]^ Research has also shown a robust negative association between meaning in life and depressive symptoms across diverse populations, supporting its relevance as a core construct in understanding psychological well-being.^[6]^

Developing a sense of meaning is crucial during adolescence, as it fosters personal growth and psychological well-being.^[7]^ Meaning in life refers to how individuals seek and find purpose or significance, and it is typically divided into 2 dimensions: the presence of meaning (PML), which reflects the perception of life as meaningful, and the search for meaning (SML), which involves actively striving to understand and create meaning.^[8]^ Both dimensions are important for adolescent mental health, particularly in relation to depressive symptoms. PML is generally inversely related to depressive symptoms, with higher meaning associated with fewer depressive symptoms.^[9,10]^ However, the relationship between SML and depressive symptoms remains inconclusive. For instance, SML has been found to negatively relate to depressive symptoms in Chinese adolescents, whereas it may positively relate to depressive symptoms in Asian American samples.^[9–11]^ Therefore, further research is needed to clarify how PML and SML are differentially associated with depressive symptoms.

In previous research, the relationship between PML, SML, and adolescent depressive symptoms has primarily been examined by operationalizing depressive symptom severity using total scores from standardized symptom scales.^[9,10]^ However, this approach does not account for the heterogeneity of depressive symptoms across individuals.^[12]^ Certain symptoms, such as sadness, loneliness, and self-hatred, may be more central and influential than others during adolescence.^[13–15]^ It remains unclear which specific depressive symptoms are directly related to PML and SML. Therefore, identifying the associations between PML, SML, and specific depressive symptoms may enhance our understanding of how different dimensions of meaning in life relate to adolescent depressive symptoms from a network perspective.

Network analysis offers granular insights by exploring the potential link between depressive symptoms and meaning in life at the symptom-specific level.^[16]^ In this approach, nodes represent individual symptoms, and edges signify the associations between them, capturing potential pathways of activation or influence as one symptom propagates its effects across the network.^[17,18]^ Based on centrality indices, nodes can also serve as central symptoms, which may exert the most influence within the entire network. A recent network analysis of depressive symptoms found that depressed affect, feelings of failure, sadness, and lethargy are central symptoms among Chinese adolescents.^[19]^ However, studies employing network analysis to investigate the interplay between meaning in life and depressive symptoms among Chinese adolescents remain limited. The network approach enables a deeper understanding of the depressive symptoms directly associated with dimensions of meaning in life, as well as their centrality within the symptom network.^[20]^ Moreover, PML or SML may alter the overall structure of depressive symptom networks in adolescents (e.g., the strength of connections among symptoms), rather than simply affecting individual symptoms.^[21]^ Given the network approach’s methodological advantages in clarifying the relationship between meaning in life and depressive symptoms in adolescents, coupled with the relative scarcity of research in this area,^[21]^ our aim is to offer a novel perspective on these relationships.

This study employed network analysis to examine the associations between meaning in life and depressive symptoms in a large sample of Chinese adolescents. Given the heterogeneity of depressive symptoms and the conceptual unity of meaning in life, depressive symptoms were analyzed at the item level, while meaning in life was analyzed at the dimensional level. Adolescence is a high-risk period for depressive symptoms and a critical stage for meaning in life development.^[5,22]^ Drawing on existentialism theory, we examined the relationships between depressive symptoms and meaning in life in this population.^[23]^ We hypothesized that both PML and SML would be negatively associated with depressive symptoms, but would exhibit distinct patterns of direct and indirect associations within the symptom network. Guided by emotion dysregulation theory, which emphasizes difficulties in managing negative emotions as a core feature of depression, we hypothesized that symptoms related to negative affect would be central in the depressive symptom network.^[24]^ Finally, we investigated whether the structure of depressive symptom networks differed across levels of PML and SML, hypothesizing that adolescents with lower PML or SML would display more densely interconnected networks of depressive symptoms.

## 2. Methods

### 2.1. Participants

A total of 2253 adolescents were recruited from elementary, middle, and high schools in 5 cities of Shandong Province, China, using a convenience sampling method. After eliminating regular responses, 2052 valid questionnaires were obtained (effective response rate: 91.07%; M_age_ = 15.00 years; SD = 1.79). Among the adolescents, 254 (12.38%) were in elementary school, 509 (24.81%) in middle school, and 1289 (62.81%) in high school. The sample consisted of 968 boys (47.20%) and 465 only children (22.66%).

The study received approval from the Research Ethics Board of the Shandong Second Medical University (Protocol Number: 2021YX027). Before enrollment, informed consent was obtained from both the parents and the adolescents, addressing the purpose of the study, confidentiality, voluntariness, and the right to withdraw at any time.

### 2.2. Measures

#### 2.2.1. *Chinese Meaning in Life Questionnaire (C‑MLQ*)

The Chinese Meaning in Life Questionnaire (C-MLQ) consists of 10 items designed to assess 2 dimensions of meaning in life: the PML and the SML.^[25]^ Responses are rated on a 7-point Likert scale, with total scores ranging from 0 to 70, where higher scores indicate higher levels of meaning in life. The Cronbach α coefficients for the PML and SML subscales were 0.66 and 0.93, respectively.

#### 2.2.2. A short Chinese version of a Center for Epidemiologic Studies Depression Scale (CESD-9)

The Center for Epidemiologic Studies Depression Scale (CESD-9) is a 9-item instrument designed to assess depressive symptoms, encompassing 3 core dimensions: depressed affect, diminished positive affect, and psychomotor retardation.^[26]^ Items are rated on a 4-point Likert scale, with total scores ranging from 0 to 27; higher scores indicate more severe depressive symptoms. Items 5 and 7 are reverse-scored according to the original scoring protocol. Participants were asked to report the frequency of symptoms experienced during the past week. The scale employs 10 and 17 as thresholds for depressive tendency and high risk, respectively.^[27]^ The Cronbach α coefficient for the scale was 0.85.

### 2.3. Exploratory analyses

All analyses were conducted using R Studio Version 4.2.2 (Posit Inc., Boston). First, multivariate outliers were identified and excluded using the Mahalanobis distance to ensure the integrity of the data for subsequent analyses. Univariate normality was assessed by examining skewness and kurtosis, with skewness values considered acceptable within the range of −2 to +2 and kurtosis values within the range of −7 to +7.^[28]^ Participants were divided into high and low PML or SML groups based on the sample mean, with scores above the mean categorized as high and scores at or below the mean categorized as low. Independent *t*-tests were performed to examine group differences in total CESD-9 scores (α = .05). Finally, to ensure that the nodes in the network analysis represented distinct constructs, redundancy was assessed using the Goldbricker function from the networktools R package (version 1.2.3).^[29]^ Redundancy was evaluated by calculating the correlations between node pairs measuring the same construct and their correlations with all other nodes. Pairs with <20% unique correlation were considered redundant, which is essential for confirming the independence of the network’s structure.^[29]^

### 2.4. Network analyses

We used the qgraph (version 1.9.4) and bootnet (version 1.5.0) R-packages to estimate and visualize the network structure via a Gaussian Graphical Model, which captures conditional dependencies between variables.^[30–32]^ The Least Absolute Shrinkage And Selection Operator algorithm was applied to regularize the model by zeroing out weaker correlations, producing a sparse and interpretable network.^[33]^ Model optimization was achieved using the Extended Bayesian Information Criterion, which refined the network structure.^[34]^ In the network analysis, the CESD-9 items and the PML and SML were represented as nodes, with edges showing pairwise associations. Thicker edges indicated stronger correlations, facilitating clear visualization of the relationships between depressive symptoms and dimensions of meaning in life.^[30]^

Centrality indices were calculated to evaluate node importance in the network, specifically strength (the sum of absolute edge weights connected to a node) using the qgraph R-package (version 1.9.4).^[31]^ Strength is a reliable metric for determining a node’s central role.^[35]^ We identified the most central nodes by computing strength through the qgraph R-package (version 1.9.4), which sums the absolute edge weights of each node, reflecting its total connectivity strength within the network. As the most widely used and empirically validated centrality measure in psychopathology research, strength demonstrates superior stability and interpretability compared to alternatives (e.g., betweenness, closeness).^[31]^ Additionally, we calculated predictability using the mgm R-package (version 1.2-13), which measures the proportion of variance in a node explained by its adjacent nodes.^[28]^

Non-parametric bootstrapping was employed to calculate edge values, and the network’s accuracy and stability were assessed using the bootnet R-package (version 1.5).^[20]^ First, the correlation stability coefficient (CS-C), derived from a case-dropping bootstrap procedure, was used to evaluate the stability of centrality indices, with Epskamp recommending a CS-C above 0.25, ideally above 0.5.^[33]^ Second, edge weight accuracy was assessed using bootstrapped 95% confidence intervals (CIs), with narrower CIs indicating a more reliable network.^[33]^ Finally, bootstrap difference tests were conducted to compare centrality indices and edge weights, using 1000 permutations to evaluate differences in network properties.^[35]^

### 2.5. Expanding the meaning in life with depressive symptoms network

#### 2.5.1. Flow network analyses of meaning in life

To identify symptoms directly linked to PML and SML, we used the flow function in the qgraph R-package (version 1.2.3).^[30]^ This approach positions PML and SML on the left and generates a vertical network to illustrate the direct and indirect relationships between depressive symptoms.

#### 2.5.2. Network comparison meaning in life

We examined differences in depressive symptom networks between adolescents with high and low scores on the PML and SML (low: <mean; high: ≥mean). Dichotomizing based on mean scores allows for comparison of the relationship between different levels of PML and SML and depressive symptoms, with higher levels serving as a protective factor and lower levels indicating greater vulnerability. This method is commonly employed in psychological research.^[36]^ The network comparison test (Version 2.2.1) was used to assess network differences in 2 indices: global strength invariance (the absolute sum of all weighted edges) and network structure invariance (the maximum difference in edge weights).^[37]^ Statistical significance was determined through 1000 permutations.^[38]^

## 3. Results

### 3.1. Preliminary results

We identified no multivariate outliers in the data. However, the CESD-9 item 1 displayed a skewness of 2.20, slightly exceeding the acceptable range, yet it was retained for analysis.^[39]^ Table 1 presents the means, standard deviation, skewness, and kurtosis for all depressive symptoms, as well as the PML and SML dimensions. The mean CESD-9 score for the sample was 4.01 (SD = 4.25), with 174 participants (8.50%) scoring above the cutoff for depressive tendencies and high risk. Independent *t*-tests revealed that adolescents with low PML and SML scores reported significantly higher levels of self-reported depressive symptoms (PML: *P* < .001; high: M = 5.63, SD = 4.72, n = 983; low: M = 2.52, SD = 3.08, n = 1069; SML: *P < *.001; high: M = 5.25, SD = 4.97, n = 1063; low: M = 2.68, SD = 2.47, n = 989). Correlation analysis indicated no redundancy among the items, as <20% of correlations were statistically significant. Consequently, all CESD-9 items and C-MLQ dimensions were included in the network analyses.

**Table 1:** Descriptive data of CESD-9 and C-MLQ.

	Mean	SD	Skewness	Kurtosis
CESD-9 1: Downcast	0.27	0.55	2.20	5.10
CESD-9 2: Attention deficit	0.43	0.66	1.53	2.10
CESD-9 3: In low spirits	0.38	0.61	1.60	2.31
CESD-9 4: Fatigue	0.32	0.58	1.84	3.32
CESD-9 5: Lack of happiness	0.79	0.95	0.98	−0.10
CESD-9 6: Loneliness	0.33	0.62	1.96	3.81
CESD-9 7: Inability to enjoy life	0.72	0.93	1.11	0.18
CESD-9 8: Sadness	0.33	0.58	1.79	3.14
CESD-9 9: Motiveless	0.44	0.67	1.52	2.03
PML	23.42	5.09	−0.71	1.35
SML	26.62	6.44	−0.89	0.92

PML = presence of meaning, SML = search for meaning.

### 3.2. Network structure and stability

**Figure 1.**
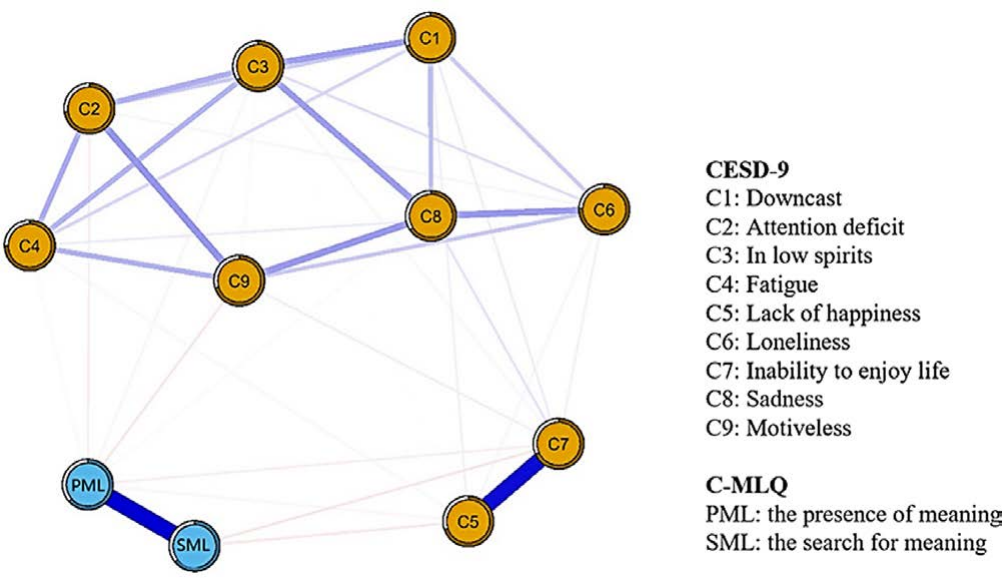
Network structures of depressive symptoms and meaning in life. Ring-shaped pie charts represent predictability. Blue edges represent positive associations between 2 nodes, while red edges represent negative associations.

Figure 1 illustrates the network analysis depicting the relationships between PML, SML, and depressive symptoms. Forty-one of the 55 edges were above zero.

Notably, all depressive symptoms showed negative correlations with both SML and PML (Table S2, Supplemental Digital Content, https://links.lww.com/MD/Q109). The predictability of nodes (shown in the ring-shaped pie charts in Fig. 1) had an average value of 0.52, indicating that neighboring nodes account for 52% of the node variation.

**Figure 2.**
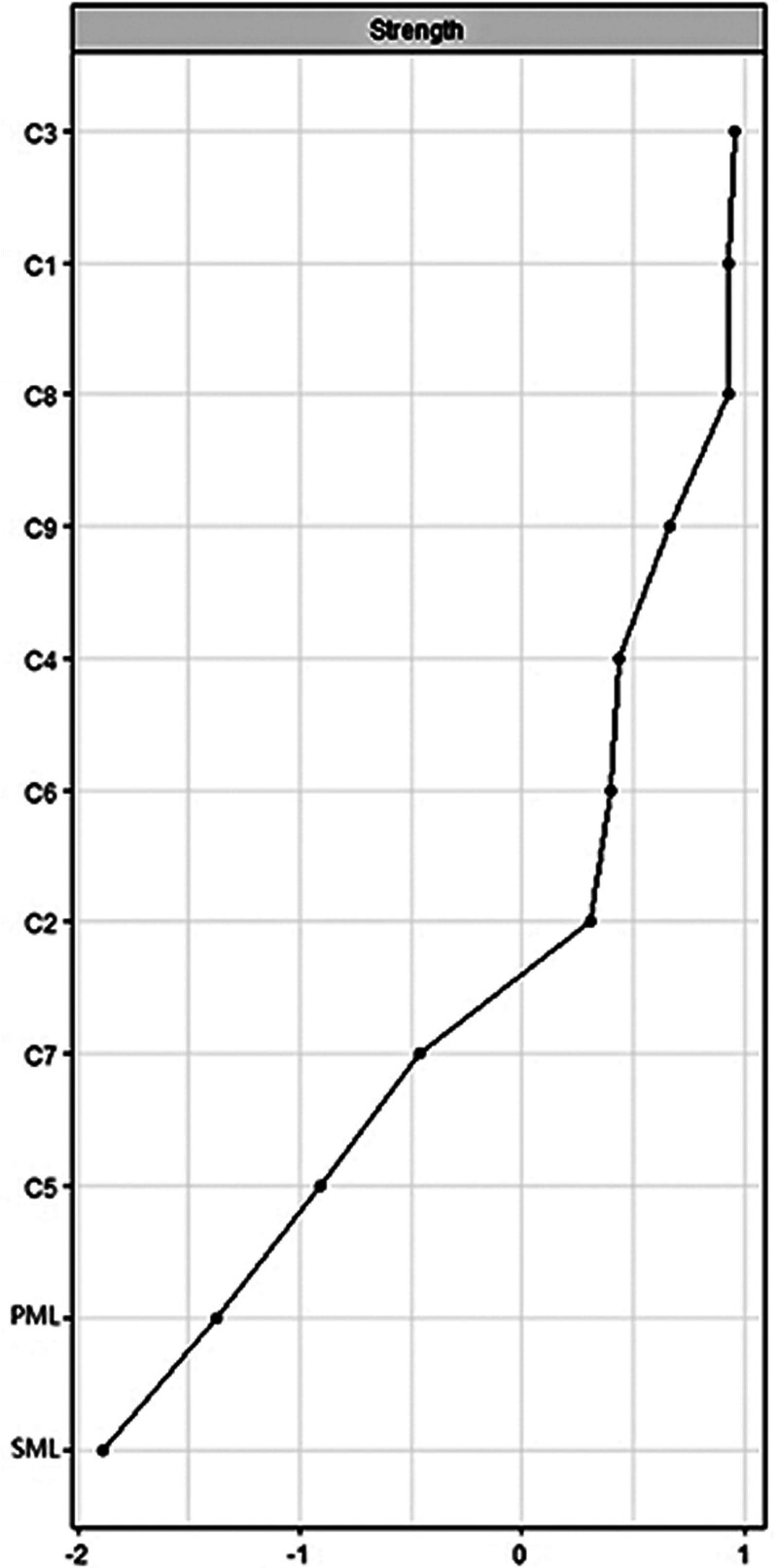
Strength of symptoms of depression and meaning in life. C1 = downcast, C2 = attention deficit, C3 = in low spirits, C4 = fatigue, C5 = lack of happiness, C6 = loneliness, C7 = inability to enjoy life, C8 = sadness, C9 = motiveless. PML = presence of meaning, SML = search for meaning.

In order to examine the relative importance of nodes in the network, we calculated node strength. Figure 2 illustrates the network strength index, and the corresponding node-specific strength values are presented in Table S1, Supplemental Digital Content, https://links.lww.com/MD/Q109. Among the CESD-9 nodes, “in low spirits (C3),” “downcast (C1),” and “sadness (C8)” exhibited the highest strength values. Figure S1, Supplemental Digital Content, https://links.lww.com/MD/Q110 shows the stability of strength, with the CS-C value being 0.75. Variations in edge weights and node strength are presented in Figs. S2 and S3, Supplemental Digital Content, https://links.lww.com/MD/Q110. Bootstrapped 95% CIs for the edges are shown in Figure S4, Supplemental Digital Content, https://links.lww.com/MD/Q110. Additionally, the network analysis and centrality indices of all items of the C-MLQ and CESD-9 are presented in Figures S5 and S6, Supplemental Digital Content, https://links.lww.com/MD/Q110.

### 3.3. Expanding the depressive symptoms network with meaning in life

#### 3.3.1. Flow network of meaning in life

**Figure 3.**
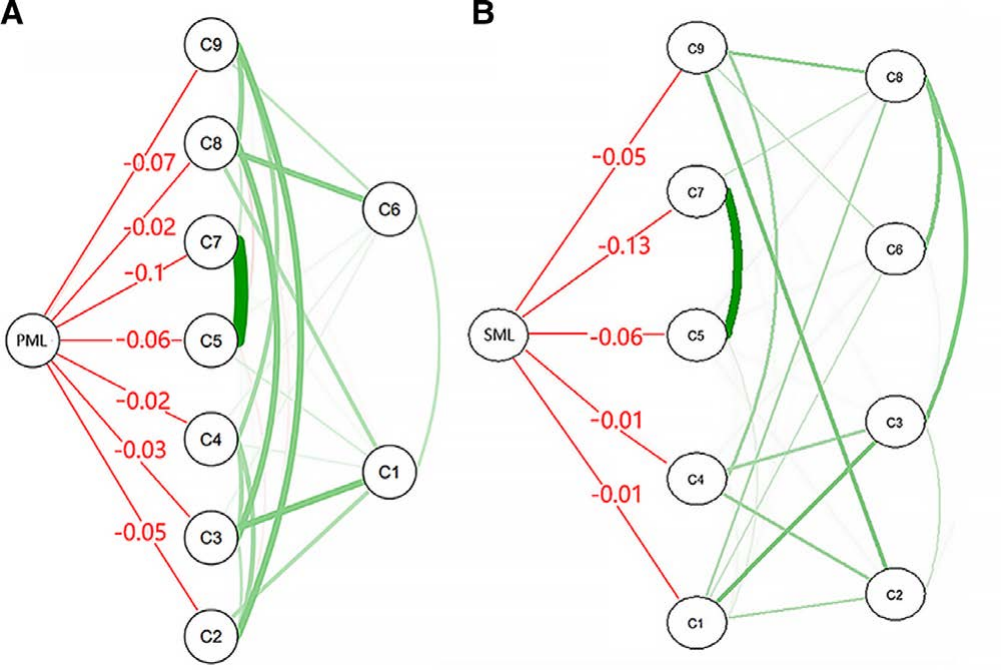
Flow network of (A) PML and (B) SML. Green edges represent positive associations between 2 nodes, while red edges represent negative associations. For clearer visualization, only symptom nodes directly linked to two dimensions were labeled with full names. C1 = downcast, C2 = attention deficit, C3 = in low spirits, C4 = fatigue, C5 = lack of happiness, C6 = loneliness, C7 = inability to enjoy life, C8 = sadness, C9 = motiveless. PML = presence of meaning, SML = search for meaning.

Figure 3A and B show the flow networks for PML and SML for directly related depressive symptoms, respectively. Depressive symptoms directly associated with PML and SML are located in the center of the figure. Among them, “lack of happiness (C5),” “inability to enjoy life (C7),” “fatigue (C4),” and “motiveless (C9)” were nodes co-connected with meaning in life.

#### 3.3.2. Networks comparison

In terms of global strength, the depressive symptom networks in the low PML group were more densely connected than those in the high PML group (*P* = .02). However, no significant difference was found in the network structure (*P* = .40). Similarly, there were no significant differences between the low and high SML groups in terms of global strength (*P* = .18) and network structure (*P* = .43). The detailed network comparison results are presented in Figures S7–S9, Supplemental Digital Content, https://links.lww.com/MD/Q110.

## 4. Discussion

This is the first study to apply a network perspective to examine the relationship between adolescent depressive symptoms and meaning in life among Chinese adolescents. Overall, both PML and SML were negatively correlated with depressive symptoms. Specifically, symptoms such as “lack of happiness (C5),” “inability to enjoy life (C7),” “fatigue (C4),” and “motiveless (C9)” were directly associated with meaning in life. The network analysis revealed that “in low spirits (C3),” “downcast (C1),” and “sadness (C8)” were the most central symptoms in the network. Notably, adolescents with lower levels of PML exhibited a denser network of depressive symptoms.

Consistent with prior research, our findings reveal that PML and SML were negatively related to depressive symptoms.^[11,12]^ Specifically, PML serves a protective role by promoting positive affect and overall mental health.^[40]^ According to the meaning maintenance model, PML helps individuals preserve a sense of purpose and coherence through a series of cognitive processes, thereby enhancing psychological resilience and counteracting negative emotions.^[41]^ The observed negative correlation between SML and depressive symptoms in Chinese adolescents contrasts with findings in Western populations. This divergence can be explained by cultural differences, as Confucianism plays a significant role in shaping Chinese values. In this context, individuals are encouraged to cultivate resilience in the face of adversity and actively seek meaning with a proactive mindset.^[42]^ For Chinese adolescents, the SML often manifests in positive adaptation and self-improvement, which are inversely related to depressive symptoms.

The flow network analysis revealed that depressive symptoms such as “lack of happiness (C5),” “inability to enjoy life (C7),” “fatigue (C4),” and “motiveless (C9)” were directly associated with meaning in life. These symptoms primarily reflect deficits in positive affect and reduced engagement in meaningful, self-directed activity, both of which are closely linked to an adolescent’s sense of meaning. Previous studies have found that individuals with a stronger meaning in life tend to experience more positive affect.^[36]^ According to the broaden-and-build theory, positive affects expand individuals’ thought-action repertoires and promote psychological flexibility.^[43]^ This broadened perspective enables adolescents to cope more effectively with stress and build enduring psychological resources, which may buffer against depressive symptoms. Similarly, from the perspective of self-determination theory, adolescents who perceive their lives as meaningful are more likely to pursue intrinsically motivated goals that fulfill core psychological needs.^[44]^ Therefore, the observed associations likely reflect how meaning in life is embedded in a broader network of affective and motivational symptoms that are linked to other depressive symptoms through affective and motivational pathways.

In addition, our analysis identified “in low spirits (C3),” “downcast (C1),” and “sadness (C8)” as the most central nodes in the depressive symptom network. This finding is consistent with previous research, which has consistently found similar symptoms to be central in both clinical and non-clinical populations.^[45,46]^ As core diagnostic features of major depressive disorder according to the DSM-5, these symptoms likely function as hubs within the network, maintaining strong connections with a range of other symptoms.^[47]^ Their centrality suggests that they may play a key role in the onset and persistence of depressive experiences, and thus represent critical targets for both prevention and intervention efforts.^[48]^ Our findings indicated that adolescents with low levels of PML exhibited more strongly interconnected networks of depressive symptoms, consistent with previous hypotheses. This suggests that while both PML and SML are negatively correlated with depressive symptoms, PML plays a more central role in influencing the interrelatedness or intensity of these symptoms. This may reflect the notion that PML has a greater impact than SML in the development of adolescent depressive symptoms.^[49]^

Our study primarily contributes to existentialist theory by demonstrating a robust connection between meaning in life and depressive symptoms. Unlike prior research that treated depression as a unitary construct, our study adopts a symptom-level perspective, revealing that meaning in life is associated with specific symptoms – particularly lack of positive affect and activity retardation. This suggests that meaning in life may exert its protective role not uniformly across all symptoms, but specifically through emotional and behavioral pathways. In doing so, our study advances theoretical understanding of how meaning in life operates within the symptom network of depression, bridging the gap between abstract existential concepts and concrete psychological manifestations.^[50,51]^ These insights may inform the development of targeted interventions that enhance meaning in life to alleviate specific depressive symptoms in adolescents.

This study holds significant practical implications. Our findings suggest a strong association between meaning in life and depressive symptoms, highlighting the importance of fostering adolescents’ sense of meaning. Educators and practitioners may consider implementing interventions that enhance meaning in life, such as meaning-making coping strategies and life review interventions.^[52,53]^ Moreover, given the close link between meaning in life and specific symptoms like reduced positive affect and activity retardation, interventions should prioritize these targets. Mindfulness-Based Cognitive Therapy has demonstrated efficacy in enhancing emotional regulation and promoting positive affect.^[54]^ Additionally, encouraging adolescents’ participation in structured activities through enrichment programs and social interactions can help restore motivation and mitigate symptoms related to activity retardation.^[55]^

This study has several limitations. First, the cross-sectional design limits the ability to infer causality between variables, highlighting the need for longitudinal research with larger sample sizes. Longitudinal follow-up studies are essential to better understand the temporal development and interactions of symptoms. Second, the reverse scored items (C5 and C7) may introduce reverse scoring bias, potentially distort the network structure and create the illusion of item isolation. Therefore, these 2 nodes should be interpreted with caution.

In this study, we used network analysis to investigate the symptom-level associations between meaning in life and depressive symptoms among Chinese adolescents. This study revealed the relations between meaning in life and depressive symptoms, lending support to existentialism theory.^[56]^ Furthermore, the meaning in life was linked to other depressive symptoms through the lack of positive affect and activity retardation. These results offer valuable insights for future research and intervention strategies aimed at addressing depressive symptoms in Chinese adolescents.

## Acknowledgments

We want to thank the participants in our study.

## Author contributions

**Conceptualization:** Xiubin Wang, Nengzhi Jiang.

**Data curation:** Xiubin Wang.

**Methodology:** Xiubin Wang.

**Project administration:** Xiubin Wang.

**Validation:** Xiubin Wang.

**Writing – original draft:** Xiubin Wang.

**Writing – review & editing:** Xiubin Wang, Nengzhi Jiang.

**Funding acquisition:** Nengzhi Jiang.

**Supervision:** Nengzhi Jiang.

## Supplementary Material


