# Supplementary material for: Network analysis of meaning in life and depressive symptoms in Chinese adolescents
Source: Medicine (Baltimore). 2025 Sep 19;104(38):e44762. doi: 10.1097/MD.0000000000044762 (PMC12459494; doi:10.1097/MD.0000000000044762)
Supplement: Supplementary file 1 [file medi-104-e44762-s001.pdf]

Table S1 The raw values of strength.

Node	Strength
C1	5.66
C2	5.08
C3	5.69
C4	5.20
C5	3.70
C6	5.17
C7	4.38
C8	5.67
C9	5.41
PML	3.52
SML	3.05

Note. C1: Downcast C2: Attention deficit C3: In low spirits C4: Fatigue C5: Lack of happiness C6: Loneliness C7: Inability to enjoy life C8: Sadness C9: Motiveless PML: presence of meaning SML: search for meaning

Table S2 Correlation matrix of the C-MLQ and CESD-9 items.

	C1	C2	C3	C4	C5	C6	C7	C8	C9	PML	SML
C1	1										
C2	0.51	1									
C3	0.64	0.54	1								
C4	0.49	0.54	0.55	1							
C5	0.29	0.17	0.27	0.21	1						
C6	0.51	0.42	0.52	0.44	0.24	1					
C7	0.31	0.23	0.30	0.23	0.70	0.27	1				
C8	0.59	0.47	0.64	0.49	0.24	0.58	0.31	1			
C9	0.48	0.59	0.51	0.54	0.23	0.49	0.27	0.57	1		
PML	-0.23	-0.23	-0.25	-0.22	-0.27	-0.21	-0.30	-0.25	-0.27	1	
SML	-0.20	-0.16	-0.18	-0.17	-0.28	-0.16	-0.31	-0.19	-0.20	0.75	1
