# Supplementary material for: Network analysis of meaning in life and depressive symptoms in Chinese adolescents
Source: Medicine (Baltimore). 2025 Sep 19;104(38):e44762. doi: 10.1097/MD.0000000000044762 (PMC12459494; doi:10.1097/MD.0000000000044762)
Supplement: Supplementary file 2 [file medi-104-e44762-s002.pdf]

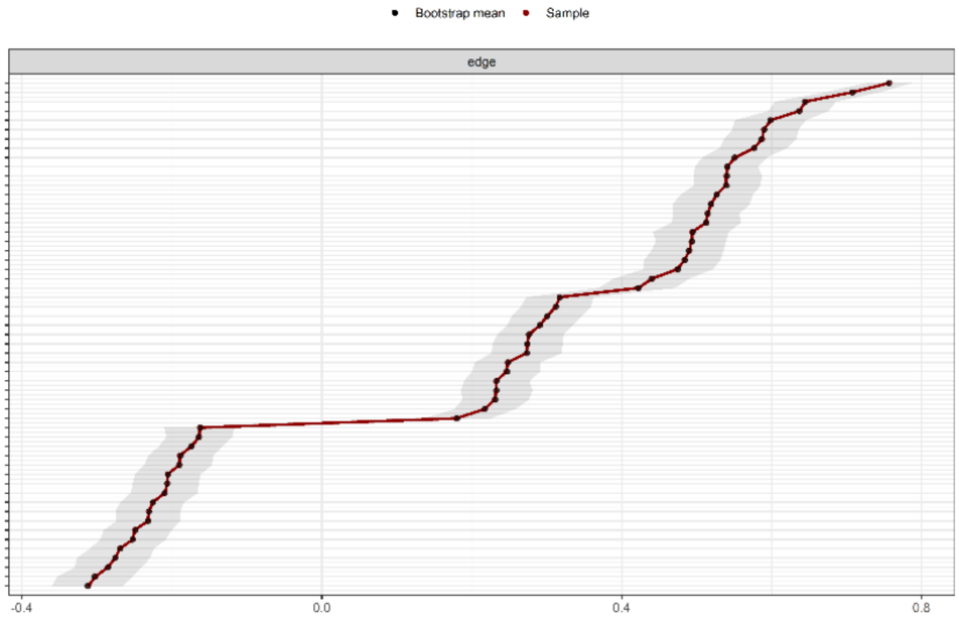

Figure S1. Confidence intervals of estimated edge weights.

Note. The red line indicates the values of each edge weight and the gray area is the 95% Confidence Interval. Each horizontal line represents one edge of the network, ordered from the edge with the highest edge weight to the edge with the lowest.

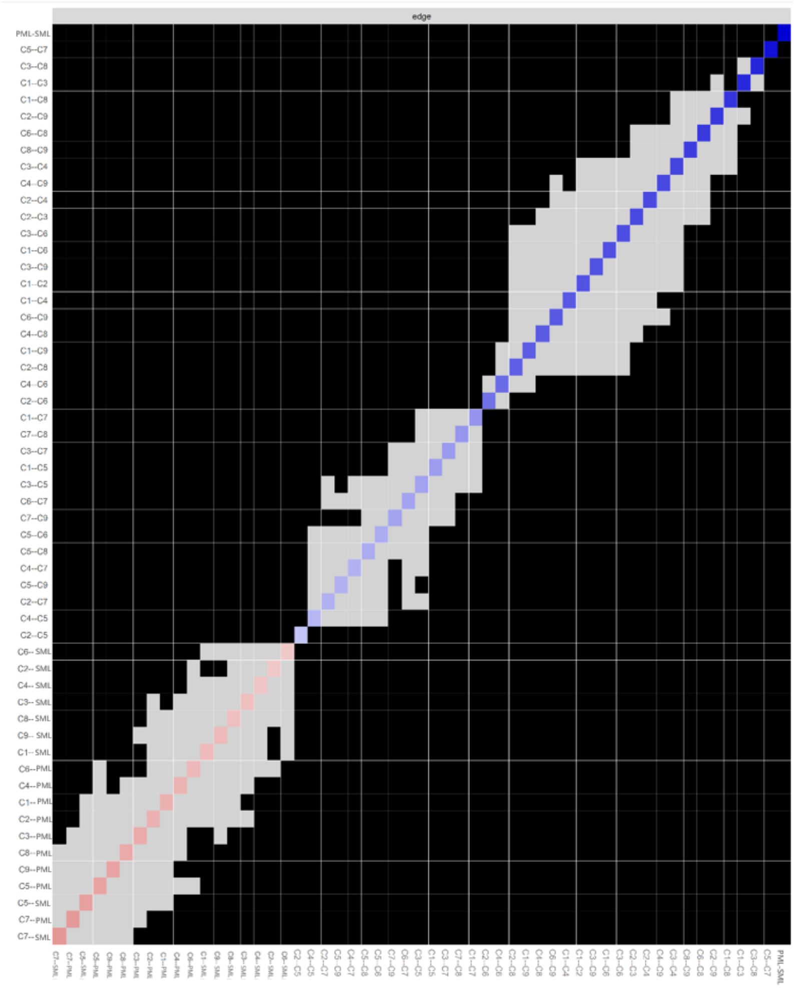

Figure S2. Edge weight difference test.

Note. Gray boxes indicate edges that do not differ significantly, and black boxes represent edges that differ significantly. Blue boxes in the edge-weight plot correspond to positive correlations.

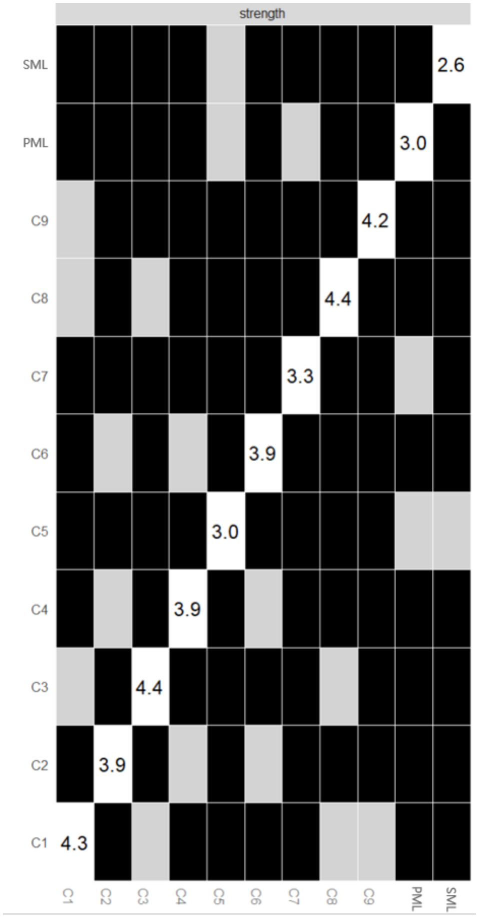

Figure S3. Node strength difference test.

Note. Gray boxes indicate edges that do not differ significantly, and black boxes represent edges that do differ significantly.

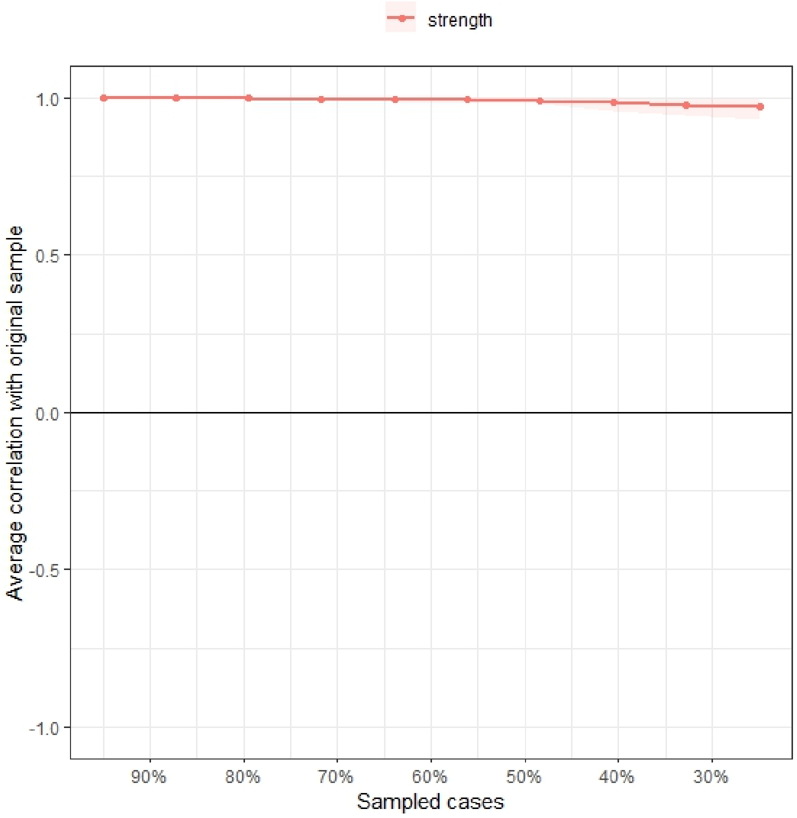

Figure S4. Stability of node strength.

Note. The x-axis represents the percentage of cases in the original sample used at each step. The y-axis represents the average correlations between the original network's centrality indices and the centrality indices in the networks that were re-estimated after dropping increasing percentages of cases. Each line indicates the correlations of strength, while areas indicate 95% CI.

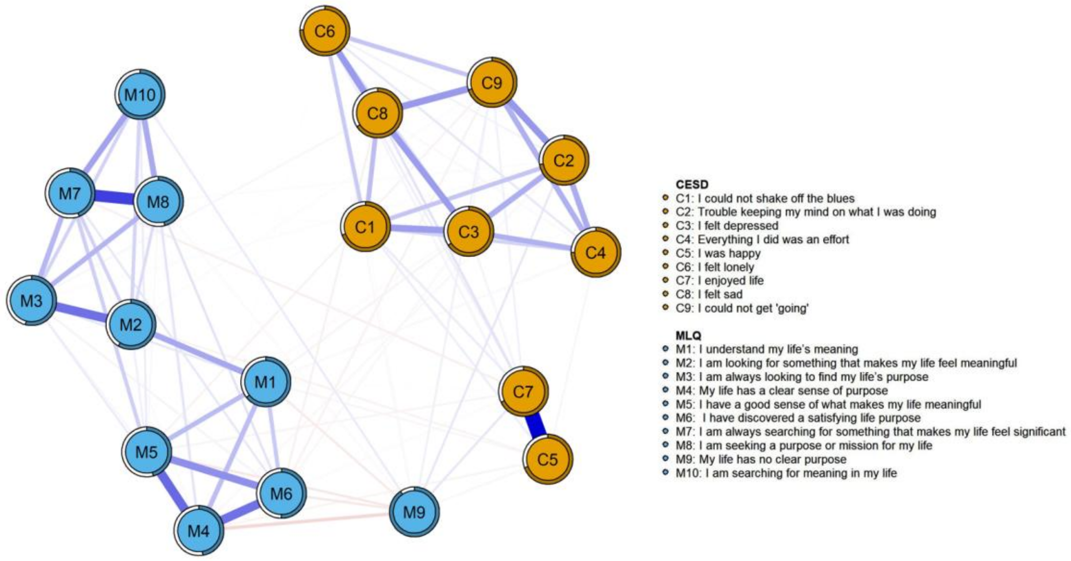

Figure S5. Network structure of depressive symptoms and items of meaning in life in Chinese adolescents. C5 and C7 are reverse scoring item.

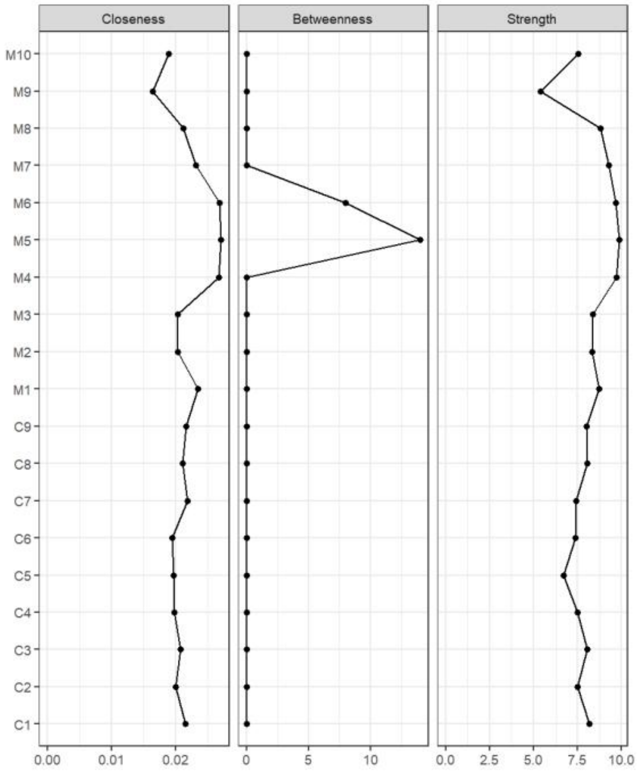

Figure S6. Centrality indices of the individual nodes.

Note. The X-axis indicates the center index.

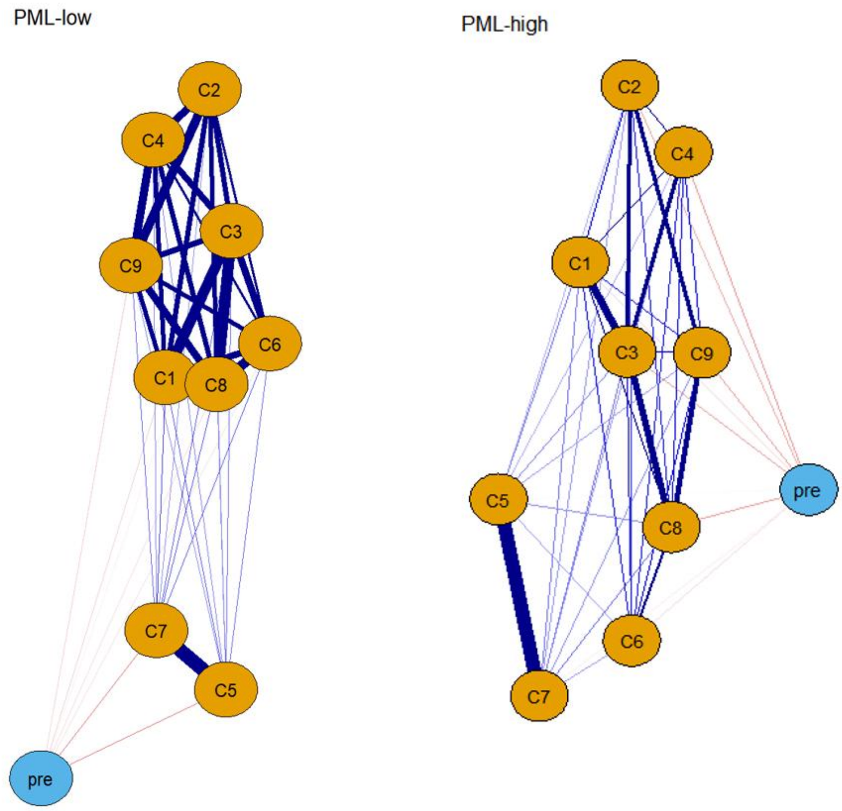

Figure S7. Network structure of depressive symptoms and low and high PML groups.
Note. The blue lines represent positive associations. The edge thickness represents the strength of the association between nodes.

The high PML group (N = 983) consisted of 439 girls (M_{age} = 14.32) and 544 boys (M_{age} = 15.03), while the low PML group (N = 1,069) included 645 girls (M_{age} = 15.12) and 424 boys (M_{age} = 14.86).

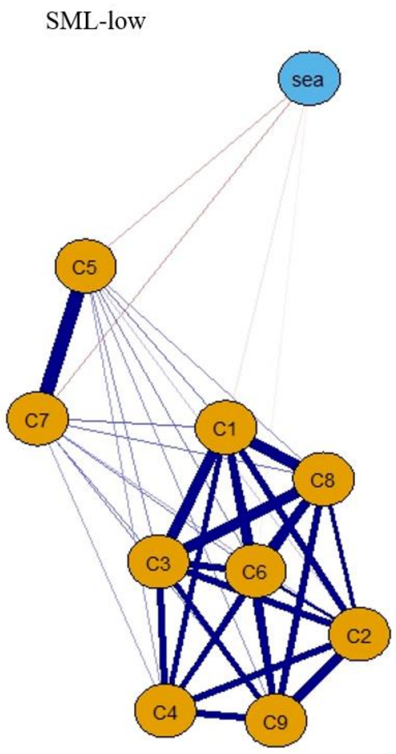

Figure S8. Network structure of depressive symptoms and low and high SML groups. *Note.* The blue lines represent positive associations. The edge thickness represents the strength of the association between nodes.

The high SML group (N = 1,063) consisted of 566 girls (M_{age} = 15.01) and 497 boys (M_{age} = 15.20), while the low SML group (N = 989) included 518 girls (M_{age} = 15.07) and 471 boys (M_{age} = 14.96).

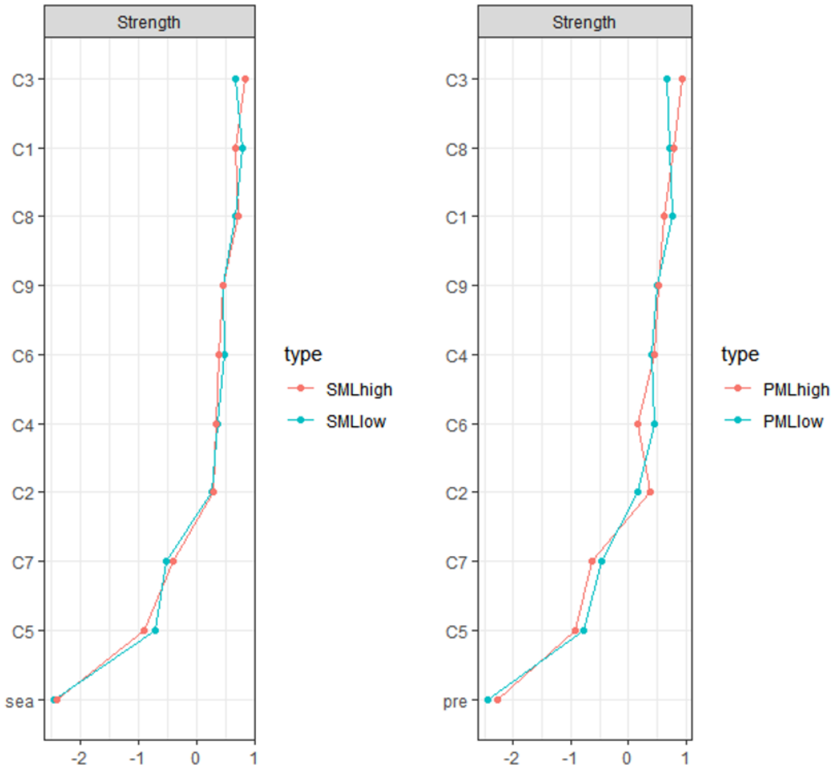

Figure S9. Centrality indices of depressive symptoms and dimensions of meaning in life.

Note. Red lines indicate high level, whereas blue lines indicate low level.
